# Nationwide trends and in-hospital outcomes of surgical versus transcatheter aortic valve replacement in Japan: a real-world analysis using administrative data

**DOI:** 10.1007/s00380-025-02640-5

**Published:** 2025-12-29

**Authors:** Yoon Kyoung Kim, Eiki Nagaoka, Kiyotoshi Oishi, Mikayo Toba, Kiyohide Fushimi, Tomoyuki Fujita

**Affiliations:** 1 Department of Surgery and Cancer, Imperial College London, South Kensington Campus, London, SW7 2AZ UK; 2 Department of Cardiovascular Surgery, Institute of Science Tokyo, M&D Tower, 1-5-45 Yushima, Bunkyo-ku, Tokyo, 113-8510 Japan; 3 Quality Management Center, Institute of Science Tokyo, M&D Tower, 1-5-45 Yushima, Bunkyo-ku, Tokyo, 113-8510 Japan; 4 Department of Health Policy and Informatics, Institute of Science Tokyo, M&D Tower, 1-5-45 Yushima, Bunkyo-ku, Tokyo, 113-8510 Japan

**Keywords:** Transcatheter aortic valve replacement/Implantation, Aortic valve replacement, Aortic stenosis

## Abstract

Aortic stenosis (AS) is the most prevalent valvular disease in developed nations. Transcatheter Aortic Valve Replacement (TAVR) offers a minimally invasive alternative to Surgical Aortic Valve Replacement (SAVR), particularly for high-risk patients. However, TAVR adoption and outcomes in Japan remain understudied due to limited national database analyses and differences in patient demographics compared to major international trials. We conducted a retrospective cohort study of aortic valve replacement (AVR) procedures from 2014 to 2021 using the National Healthcare Reimbursement database. In total, 103,076 cases were analyzed, with 33,881 undergoing TAVR and 31,337 undergoing SAVR for aortic stenosis. TAVR patients were older (median age 85 vs. 76 years, *p* < 0.0001) and more often female (66.7% vs. 51.4%, *p* < 0.0001). TAVR was associated with lower in-hospital mortality (0.88% vs. 2.4%, *p* < 0.0001), shorter hospital stays (14 vs. 24 days, *p* < 0.0001), but significantly higher medical costs (¥5,303,722 vs. ¥3,945,622, *p* < 0.0001). Among patients younger than 75 years, no significant difference in mortality was observed. In this nationwide administrative database, TAVR was associated with lower in-hospital mortality and shorter hospital stays compared to SAVR, though at a higher cost. While patient backgrounds and clinical risks could not be fully adjusted due to data limitations, the observed differences in in-hospital outcomes between TAVR and SAVR reflect real-world practice patterns. These findings should be interpreted as descriptive, not causal. The absence of a mortality difference in patients under 75 years highlights the need for further evaluation of TAVR’s long-term effectiveness and cost-efficiency in younger populations.

**Supplementary Information:**

The online version contains supplementary material available at 10.1007/s00380-025-02640-5.

## Background

### Introduction

Aortic stenosis (AS), primarily caused by age-related calcification and tissue remodeling, is the most common valvular disease in developed nations [1]. In 2019, an estimated 9.4 million people globally were affected by calcific aortic valve disease [1, 2]. In Japan, AS affects < 1% of people under 70, but over 7% of those aged ≥ 80. With Japan’s aging population, its prevalence is expected to rise [3–6].

Symptomatic AS carries a 2% monthly mortality rate, with 80% of untreated patients dying within three years [7, 8]. Early surgical aortic valve replacement (SAVR) improves survival, even in asymptomatic patients under 80 [9, 10] The role of transcatheter aortic valve replacement (TAVR) in asymptomatic patients is under investigation [10–13]. No pharmacologic therapy has yet been shown to slow or reverse AS progression [14].

TAVR, introduced in 2002, offers a less invasive alternative to SAVR [15] Initially approved for inoperable or high-risk patients, it is now used across risk levels, with trials showing non-inferiority or superiority to SAVR in 2-, 5-, and 10-year outcomes [16–23].

However, TAVR has limitations, including unknown valve durability beyond 10 years. Global guidelines recommend TAVR primarily for patients over 75 [24, 25]. Japanese guidelines suggest a flexible age cutoff of 75–80 due to longer life expectancy [26].

TAVR complications include structural valve degeneration (SVD), paravalvular leak (PVL), and higher pacemaker implantation rates [27, 28]. It is less suitable for patients with bicuspid valves, poor vascular access, or severe calcifications—scenarios where SAVR remains necessary [29].

Recent improvements in SAVR, such as enhanced recovery and minimally invasive techniques, have improved outcomes [30–32]. Still, it requires open-heart surgery with cardiopulmonary bypass [33–37].

Economic considerations are especially relevant in Japan’s aging society [38, 39]. Tools like the Diagnosis Procedure Combination (DPC) system and Health Technology Assessment (HTA) evaluate cost-effectiveness and help manage healthcare spending [40–43].

TAVR costs remain substantially higher, with kits priced around ¥4.5 million versus ¥984,000 for surgical valves [44, 45]. Cost-effectiveness analyses are mixed, highlighting the need for further evaluation across patient groups and healthcare systems [44–48].

### Study rationale

Despite favourable data from randomized controlled trials (RCTs), questions remain about TAVR’s broader application, in younger, lower-risk populations. Observational studies and registry data are essential to complement RCT findings and assess real-world outcomes [49–51].

This study aims to fill this gap by evaluating the adoption of TAVR in Japan since its approval in 2013 and comparing in-hospital mortality, total medical costs, and length of stay between SAVR and TAVR across age groups. The findings will provide valuable insights for optimizing AVR strategies globally.

## Methods

### Data source and study design

This retrospective cohort study utilized the Diagnosis Procedure Combination (DPC) database, which compiles detailed administrative and clinical data from approximately 1,757 hospitals with 483,180 beds, covering nearly all acute inpatient care in Japan [40, 41]. The DPC-based Per-Diem Payment System (DPC-PDPS) combines a bundled payment model with fee-for-service components, enabling detailed tracking of medical costs and resource allocation [40]. The database includes comprehensive patient information such as demographics, principal diagnoses, secondary conditions, complications during hospitalization, prescribed medications, and performed procedures [52]. Diagnoses and conditions were coded using the International Classification of Diseases and Related Health Problems, 10th Revision (ICD-10) [40, 41]. Online Resource provides detailed coding information.

This study adhered to the Strengthening the Reporting of Observational Studies in Epidemiology (STROBE) guidelines for cohort studies [53]. Data is available upon reasonable request to the corresponding author.

### Inclusion and exclusion criteria

Patients registered in the DPC database from January 2014 to December 2021 who underwent TAVR or SAVR for non-rheumatic aortic stenosis (AS) were included. Procedures were identified using DPC procedure codes K5551, K555-2 (TAVI310), and K555-22 (TAVI950), with TAVI950 being an updated code since 2018. AS was defined using the ICD-10 code I350. Patients aged 18 to 102 years were included, while those with incomplete data, hospitalizations not primarily for AS, or concomitant cardiac surgeries were excluded. Rheumatic AS cases were excluded due to their distinct etiology, demographics, and management, which differ significantly from non-rheumatic AS.

### Covariates and outcomes

Covariates included age, sex, and procedure type. From the 47 available DPC variables (see Online Resource), the primary outcomes were:

- In-hospital mortality: Defined using numeric discharge codes 6 and 7.
- Total medical costs: Cumulative hospitalization charges, including procedural, stay, and treatment costs.
- Length of stay: Number of days from admission to discharge.

Secondary analyses stratified outcomes by age group and year of procedure. Age-based comparisons assessed variations in mortality, cost, and length of stay. Temporal trends from 2014 to 2021 were also analyzed to evaluate procedural adoption and outcome evolution.

### Statistical analysis

Data management and cleaning were performed in Microsoft Excel. Patients were categorized by procedure type, age group, and year, and filtered based on primary and most resource-intensive diagnoses. Statistical analysis was performed using GraphPad Prism (v10.2.3). The Shapiro-Wilk test assessed normality. Continuous variables were reported as medians with interquartile ranges (IQRs) and compared using the Mann-Whitney U test. Categorical variables were compared using the Chi-squared test. A p-value < 0.05 was considered statistically significant.

### Ethical considerations

The study protocol was approved by the Institutional Review Board of Institute of Science Tokyo (Approval number: M2000-788). This study was conducted in accordance with the Declaration of Helsinki and approved by the appropriate institutional ethics committee. Patient consent was waived due to the retrospective nature of the study and the use of anonymized data. Patient identification was anonymized at the hospital level using coding equations and centralized by the Ministry of Health, Labour and Welfare. Patients retained the option to opt out of having their data included in the DPC database.

## Results

**Fig. 1:**
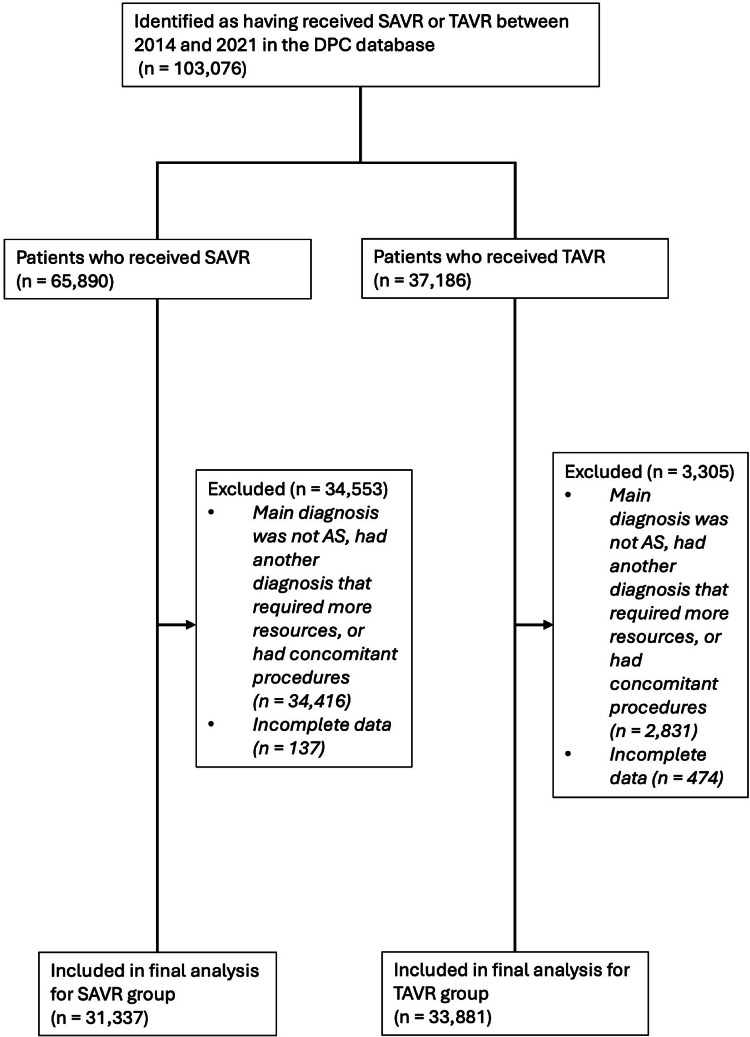
Patient Selection Flowchart. This flowchart illustrates the selection process of patients from the DPC database for the study. A total of 103,076 patients were identified as having received SAVR or TAVR between 2014 and 2021. Of these, 65,890 patients received SAVR and 37,186 received TAVR. Exclusion criteria included patients whose main diagnosis was not AS, those with another diagnosis requiring more resources or concomitant procedures, and those with incomplete data. The final analysis included 31,337 SAVR patients and 33,881 TAVR patients. *Abbreviations*: DPC – Diagnosis Procedure Combination; AS – Aortic Stenosis; SAVR – Surgical Aortic Valve Replacement; TAVR – Transcatheter Aortic Valve Replacement

Of the 103,076 patients who underwent aortic valve replacement (AVR) in the DPC database between 2014 and 2021, 31,337 met inclusion criteria for SAVR and 33,881 for TAVR (Fig. 1). The median age of the overall cohort was 81 years (IQR 75–85). TAVR patients were significantly older than those undergoing SAVR (85 vs. 76 years, *p* < 0.0001) and more likely to be female (66.7% vs. 51.4%, *p* < 0.0001). TAVR patients had lower BMI (22.4 vs. 23.1) and BSA (1.44 vs. 1.54; both *p* < 0.0001; Table 1).

**Table 1:** Characteristics of the cohort

	Overall (*n* = 65,218)	SAVR (*n* = 31,337)	TAVR (*n* = 33,881)	*p*-value
Age (years)	81[75,85]	76 [70,80]	85 [81,88]	< 0.0001
BMI	22.7 [20.4,25.3]	23.1 [20.8,25.7]	22.4 [20.0,24.9]	< 0.0001
BSA	1.45 [1.36,1.63]	1.544 [1.42,1.68]	1.44 [1.32,1.57]	< 0.0001
Center volume	495 [355,658]	460 [333,605]	525 [397,693]	< 0.0001
Days from admission to procedure (days)	4 [2,7]	4 [2,7]	3 [2,7]	< 0.0001
Female Sex (%)	59.3 (38,696/65218)	51.4 (16,112/31,337)	66.7 (22,584/33,881)	< 0.0001
In-Hospital Mortality (%)	1.6 (1,050/65,218)	2.4( 752/31,337)	0.88 (298/33,881)	< 0.0001
Length of stay (days)	19 [13,28]	24 [19,33]	14 [10,21]	< 0.0001
Total Medical Costs (JPY)	4,857,568 [3,966,496; 5,370,271]	3,945,622 [3,611,509; 4,440,920]	5,303,722 [5,075,835; 5,567,127]	< 0.0001

This table presents the baseline characteristics of patients undergoing SAVR and TAVR from the dataset. include age, female sex percentage, BMI, and BSA. Data are presented as the median [interquartile range (IQR) or as a percentage. P-values for comparisons between SAVR and TAVR groups are shown. *SAVR – Surgical Aortic Valve Replacement; TAVR – Transcatheter Aortic Valve Replacement; BMI – Body Mass Index; BSA – Body Surface Area*

**Fig. 2:**
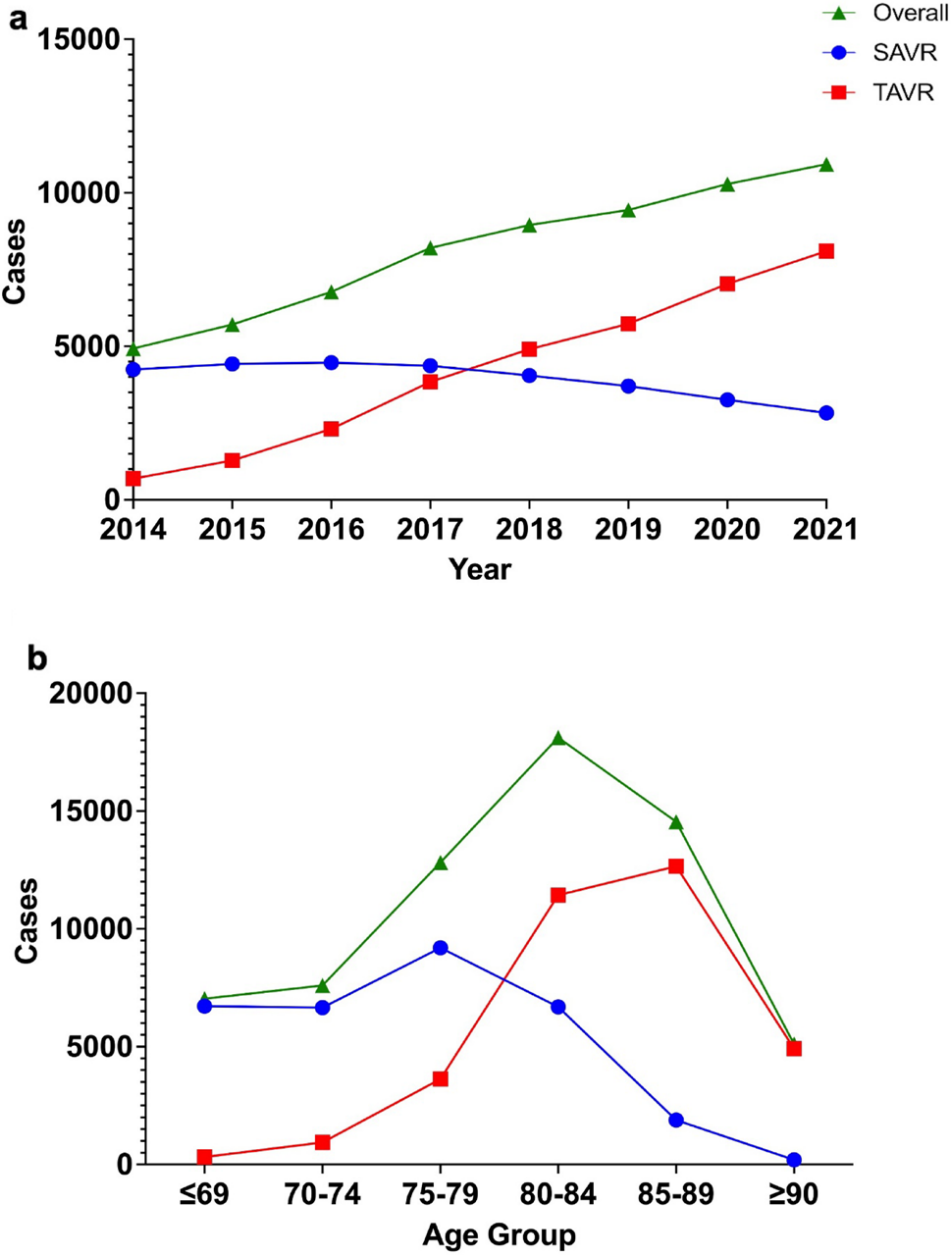
Trends in Aortic Valve Replacement Procedures. (**a**) Annual Number of Aortic Valve Replacement Cases: Displays the annual number of SAVR, TAVR, and total AVR cases from 2014 to 2021. (**b**) Distribution by Age Group: Shows the distribution of AVR cases across age groups over the study period. *Abbreviations*: AVR – Aortic Valve Replacement; SAVR – Surgical Aortic Valve Replacement; TAVR – Transcatheter Aortic Valve Replacement

**Fig. 3:**
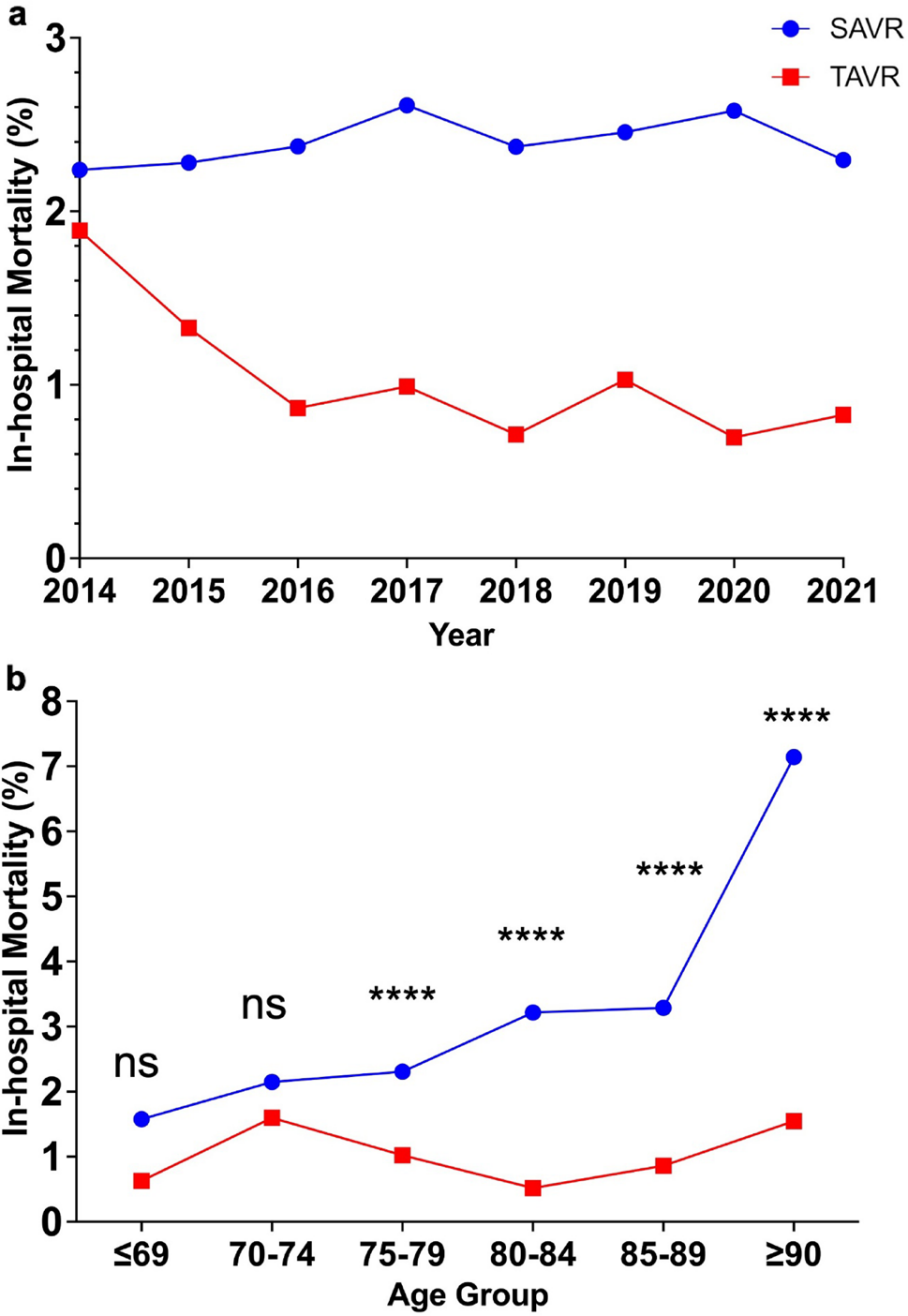
In-Hospital Mortality Rates for SAVR and TAVR. (**a**) Annual In-Hospital Mortality Rates: Trends in in-hospital mortality rates for SAVR and TAVR from 2014 to 2021. TAVR shows a consistent decline over the years. (**b**) Mortality by Age Group: In-hospital mortality rates for SAVR and TAVR categorized by age group. Mortality is significantly higher in older age groups, particularly for SAVR in the ≥ 90 age group

**Fig. 4:**
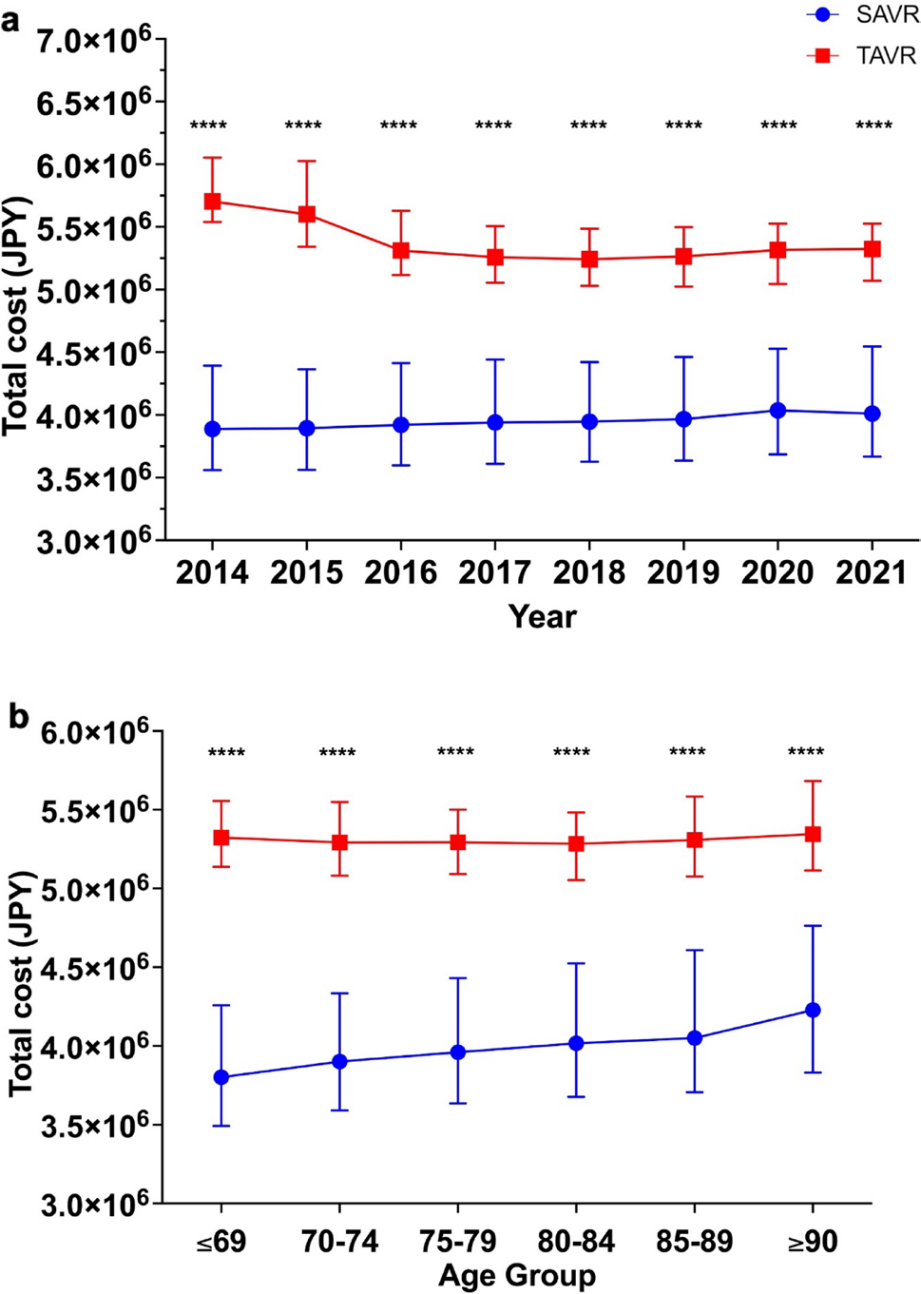
Total Medical Costs for SAVR and TAVR. (**a**) Annual Costs: Median total medical costs for SAVR and TAVR from 2014 to 2021 (JPY). TAVR consistently shows higher costs, with error bars indicating IQR. (**b**) Costs by Age Group: Median total costs for SAVR and TAVR by age group (JPY). TAVR costs are consistently higher across all age groups

**Fig. 5:**
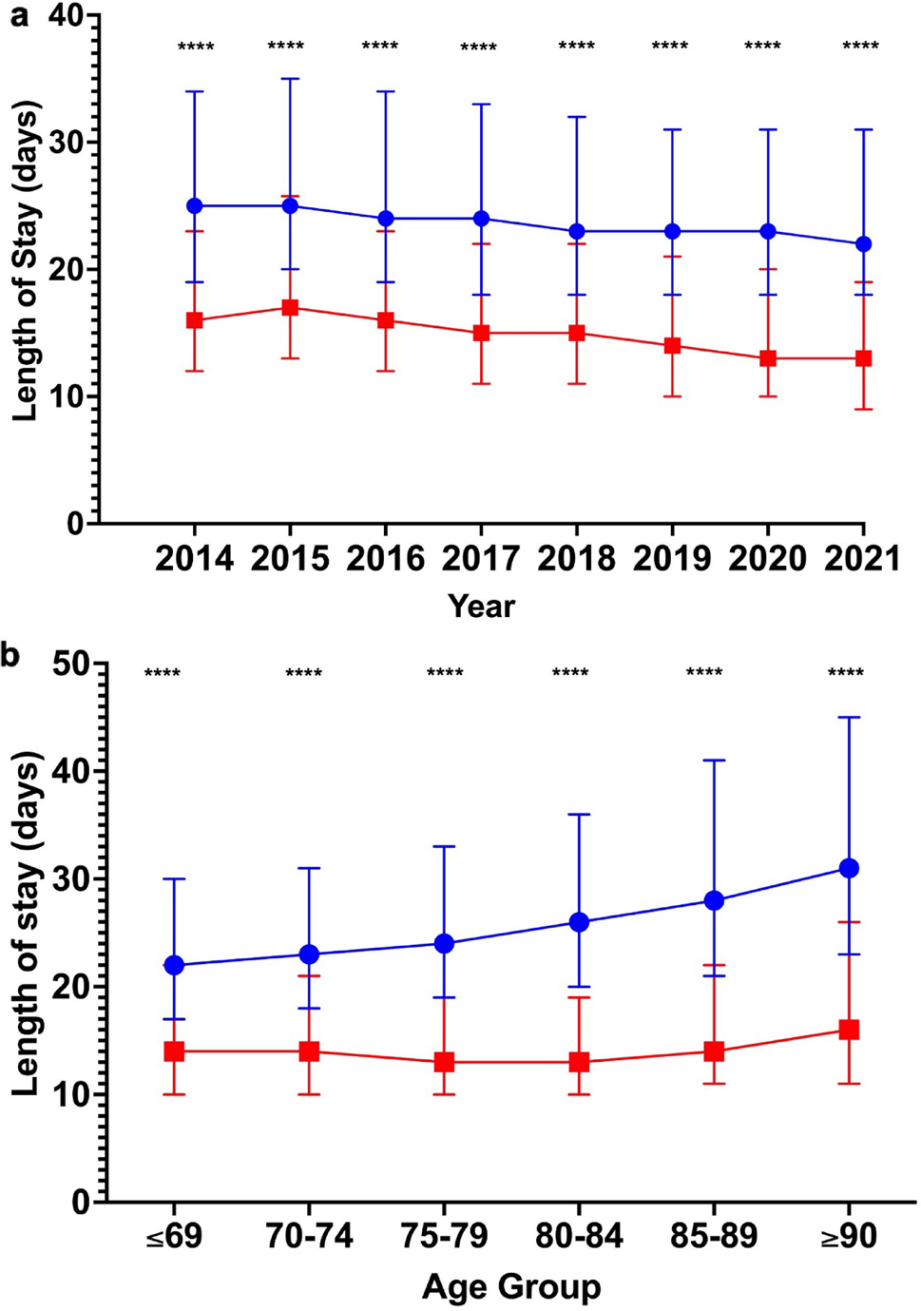
Length of Hospital Stay for SAVR and TAVR. (**a**) Annual Length of Stay: Median length of stay for SAVR and TAVR patients from 2014 to 2021 (days). TAVR shows consistently shorter stays, with error bars indicating IQR. (**b**) Length of Stay by Age Group: Median length of stay for SAVR and TAVR patients by age group, showing shorter stays for TAVR across all groups

TAVR showed lower in-hospital mortality compared to SAVR (0.88% vs. 2.4%, *p* < 0.0001). This mortality benefit was significant in patients ≥ 75 years, but not in younger groups (≤ 74 years; Tables 2 and 3). In younger cohorts, TAVR use was limited (≤ 69 years: 318 TAVR vs. 6,721 SAVR). Median hospital stay was shorter for TAVR across all age groups (14 vs. 24 days, *p* < 0.0001), while median costs were higher (¥5,303,722 vs. ¥3,945,622; ~$46,070 vs. $34,280; *p* < 0.0001). From 2014 to 2021, TAVR mortality declined from 1.9% to 0.83%, while SAVR mortality remained stable (~ 2.2–2.3%) (Figs. 2 and 3a). TAVR consistently showed shorter stays and higher costs annually and across all age brackets (Figs. 4 and 5).

**Table 2:** Outcomes of surgical aortic valve replacement (SAVR) and transcatheter aortic valve replacement (TAVR) by age group

Column1	Overall (*N* = 65,218)	SAVR (*N* = 31,337)	TAVR (*N* = 33,881)	*p*-value
In-Hospital Mortality (n/N)	1.6 (1,050/65,218)	2.4 (752/31,337)	0.88 (298/33,881)	< 0.0001
≤ 69		1.6 (106/6721)	0.63 (2/318)	0.1789
70–74		2.1 (143/6657)	1.6 (15/940)	0.2666
75–79		2.3 (212/9192)	1.0 (37/3625)	< 0.0001
80–84		3.2 (215/6685)	0.52 (59/11429)	< 0.0001
85–89		3.3 (62/1886)	0.86 (109/12656)	< 0.0001
≥ 90		7.1 (14/196)	1.5 (76/4913)	< 0.0001
Length of stay (days)	19 [13,28]	24 [19,33]	14 [10,21]	< 0.0001
≤ 69		22	14	< 0.0001
70–74		23	14	< 0.0001
75–79		24	13	< 0.0001
80–84		26	13	< 0.0001
85–89		28	14	< 0.0001
≥ 90		31	16	< 0.0001
Total Medical Costs (JPY)	4,857,568 [3,966,496; 5,370,271]	3,945,622 [3,611,509; 4,440,920]	5,303,722 [5,075,835; 5,567,127]	< 0.0001
≤ 69		3,801,201	5,323,373	< 0.0001
70–74		3,900,983	5,291,860	< 0.0001
75–79		3,959,901	5,292,772	< 0.0001
80–84		4,016,800	5,283,731	< 0.0001
85–89		4,049,944	5,307,319	< 0.0001
≥ 90		4,228,355	5,345,172	< 0.0001

This table presents the outcomes of patients undergoing SAVR and TAVR from the dataset, categorised by age groups. Outcomes include in-hospital mortality rates, length of hospital stay, and total medical costs in JPY. In-hospital mortality is presented as a percentage, with the number of deaths and the total number of patients in each age group shown in parentheses [e.g., 1.6% (1,050/65,218)]. Length of stay and total medical costs are shown as medians with interquartile ranges (IQR) for the overall SAVR and TAVR groups, but not for individual age groups. *P*-values for Chi-squared tests between SAVR and TAVR groups are shown for in-hospital mortality. *P*-values for Mann-Whitney U tests between SAVR and TAVR groups are shown for length of stay and total medical costs. *SAVR – Surgical Aortic Valve Replacement; TAVR – Transcatheter Aortic Valve Replacement; JPY – Japanese Yen.*

**Table 3:** Outcomes of surgical aortic valve replacement (SAVR) and transcatheter aortic valve replacement (TAVR) by year

Column1	Overall (*N* = 65,218)	SAVR (*N* = 31,337)	TAVR (*N* = 33,881)	*p*-value
In-Hospital Mortality (%)	1.6 (1,050/65,218)	2.4 (752/31,337)	0.88 (298/33,881)	< 0.0001
2014		2.2 (95/4242)	1.9 (13/699)	0.561
2015		2.3 (101/4428)	1.3 (17/1280)	0.035
2016		2.4 (106/4464)	0.87 (20/2310)	< 0.0001
2017		2.6 (114/4365)	0.99 (38/3838)	< 0.0001
2018		2.4 (96/4046)	0.71 (35/4905)	< 0.0001
2019		2.5 (91/3706)	1.0 (59/5732)	< 0.0001
2020		2.6 (84/3255)	0.70 (49/7030)	< 0.0001
2021		2.3 (65/2831)	0.83 (67/8098)	< 0.0001
Length of stay (days)	19 [13,28]	24 [19,33]	14 [10,21]	< 0.0001
2014		25	16	< 0.0001
2015		25	17	< 0.0001
2016		24	16	< 0.0001
2017		24	15	< 0.0001
2018		23	15	< 0.0001
2019		23	14	< 0.0001
2020		23	13	< 0.0001
2021		22	13	< 0.0001
Total Medical Costs (JPY)	4,857,568 [3,966,496; 5,370,271]	3,945,622 [3,611,509; 4,440,920]	5,303,722 [5,075,835; 5,567,127]	< 0.0001
2014		3,887,784	5,703,163	< 0.0001
2015		3,894,840	5,600,737	< 0.0001
2016		3,921,035	5,311,489	< 0.0001
2017		3,940,650	5,258,438	< 0.0001
2018		3,946,769	5,241,754	< 0.0001
2019		3,966,800	5,263,670	< 0.0001
2020		4,035,670	5,315,944	< 0.0001
2021		4,011,506	5,324,514	< 0.0001

This table presents the outcomes of patients undergoing SAVR and TAVR from the dataset over different years. Outcomes include in-hospital mortality rates, length of hospital stay, and total medical costs in JPY. In-hospital mortality is presented as a percentage, with the number of deaths and the total number of patients in each year shown in parentheses [e.g., 1.6% (1,050/65,218)]. Length of stay and total medical costs are shown as medians with interquartile ranges (IQR) for the overall SAVR and TAVR groups, but not for individual years. P-values for Chi-squared tests between SAVR and TAVR groups are shown for in-hospital mortality. *P*-values for Mann-Whitney U tests between SAVR and TAVR groups are shown for length of stay and total medical costs. *SAVR – Surgical Aortic Valve Replacement; TAVR – Transcatheter Aortic Valve Replacement; JPY – Japanese Yen*

TAVR volume increased steadily, overtaking SAVR in 2017. By 2021, TAVR cases reached 8,098 compared to 4,046 SAVR cases. Overall AVR volume increased throughout the study period (Fig. 2a). AVR distribution by age showed that SAVR was more common in patients ≤ 79 years, while TAVR predominated ≥ 80 years. The highest TAVR volume was in patients aged 85 to 89 years (Fig. 2b).

In-hospital mortality increased with age in both procedures. TAVR’s highest mortality rate occurred in the 70–74 age group (1.6%), while SAVR peaked in ≥ 90-year-olds (Fig. 3b). TAVR retained a mortality benefit over SAVR in older age groups (*p* < 0.0001). Cost analysis confirmed TAVR’s higher expenses across all years and age groups (Fig. 4). Median length of stay remained shorter for TAVR consistently (Fig. 5).

## Discussion

This nationwide analysis demonstrates that TAVR rapidly overtook SAVR in Japan by 2017. Consistent with international experience, TAVR recipients were older and had markedly shorter hospitalizations (median 10 days). The apparent mortality advantage among elderly TAVR patients should, however, be interpreted cautiously, as administrative data cannot fully account for clinical selection factors that may have excluded higher-risk elderly patients from surgery. This pattern of reduced early morbidity with TAVR is supported by other Japanese studies reporting lower rates of complications such as postoperative acute kidney injury [54]. Importantly, this mortality benefit was not observed among patients under 75 years of age.

Reimbursement levels under Japan’s National Health Insurance system remained essentially stable during the study period, with only marginal increases in procedural and device fees. For instance, TAVR procedural fees increased only from ¥374,300 in 2014 to ¥390,600 in 2022, while device reimbursements rose modestly from ¥4,430,000 to ¥4,510,000 for balloon-expandable valves and from ¥3,670,000 to ¥3,740,000 for self-expandable valves [55]. As DPC-based costs mirror these reimbursement levels, the observed economic trends likely reflect genuine shifts in practice patterns rather than artefacts of coding or pricing.

TAVR patients were predominantly female (66.7%), differing from male-dominant RCT cohorts [21, 23]. This may reflect referral patterns or anatomical differences, underscoring the importance of gender-specific research. Japan’s longer female life expectancy (87 years vs. 81 for men) reinforces the relevance of this disparity [56–60]. Compared with US cohorts, Japanese patients were older, carried higher surgical risk scores, and were predominantly female, yet paradoxically achieved lower short-term mortality and morbidity [61]. These favorable results likely reflect Japan’s stringent TAVR certification and proctoring requirements [62]. Additionally, the smaller body size of Japanese patients may have contributed to favorable outcomes, though this requires further investigation.

The expansion of TAVR to younger patients, however, presents distinct challenges. Bicuspid aortic valve (BAV) anatomy, more common in this demographic, can increase the risk of complications such as paravalvular leak (PVL) and valve migration [29, 63–66]. Importantly, younger age does not necessarily confer increased procedural safety with TAVR, particularly in patients with complex aortic anatomy. Furthermore, surgical explantation following failed TAVR is technically complex and carries substantial risk [67]. In this context, SAVR—augmented by minimally invasive techniques and sutureless prostheses—remains an important option for selected younger patients [34–37].

Our findings underscore the need for a comprehensive national registry in Japan to capture long-term outcomes and cost-effectiveness data for TAVR and SAVR. The value of such large-scale, real-world data is increasingly recognized, with recent studies from the Japanese Nationwide Registry providing critical insights into one-year survival and outcomes in specific TAVR patient subgroups, such as those with significant mitral regurgitation [68] Japan’s mandatory TAVI registry, a prerequisite for institutional accreditation, ensures comprehensive case capture [69]. Yet data entry remains decentralized and long-term follow-up is limited, constraining durability and cost-effectiveness analyses. Linking this registry with the DPC database, and harmonizing with international registries, would strengthen evidence for both clinical practice and health technology assessments.

### Strengths and limitations

This study leverages a large, standardized national database encompassing over 100,000 patients and 8 years of longitudinal data. The use of national reimbursement records ensures consistent and reliable cost data. The scale and detail of this dataset enhance its generalizability to the Japanese population. Several limitations must be acknowledged. The primary limitation of this study is the use of an administrative database, which precludes detailed risk adjustment. The absence of granular clinical data, such as the Society of Thoracic Surgeons (STS) score, EuroSCORE, heart failure severity, or anatomical contraindications, prevents a direct comparison of risk profiles between the TAVR and SAVR cohorts. This introduces a significant risk of confounding by indication, and therefore, the observed differences in mortality cannot be used to definitively conclude that one procedure is safer than the other. Additionally, reliance on pre-coded data introduces potential selection and reporting biases. Finally, the lack of long-term follow-up data restricts conclusions on durability and late outcomes of TAVR. Despite these limitations, the large sample size and real-world setting provide valuable insights into TAVR and SAVR outcomes in Japan.

## Conclusion

This study underscores the growing role of TAVR in Japan, particularly among older and high-risk patients, while raising critical questions about its application in younger populations. Addressing long-term durability, cost-effectiveness, and patient-centered outcomes will be vital. Future efforts should prioritize robust registries, international collaboration, and shared decision-making frameworks to optimize AVR strategies.

## Supplementary Information

Below is the link to the electronic supplementary material.

Supplementary Table 1. Variables extracted from DPC database. Table listing variables available to be extracted from DPC database for this study. Some coded columns did not have a decoding scheme and could not be accessed or utilised for this study.

## Data Availability

The deidentified participant data will be shared upon reasonable request. Requests for data access should be directed to the corresponding author, Dr. Eiki Nagaoka (email: nagaoka.cvsg@tmd.ac.jp). The data available includes patient demographics, procedure type, in-hospital outcomes, medical costs, and length of stay.
