# Supplementary material for: Nationwide trends and in-hospital outcomes of surgical versus transcatheter aortic valve replacement in Japan: a real-world analysis using administrative data
Source: Heart Vessels. 2025 Dec 29;41(6):403–13. doi: 10.1007/s00380-025-02640-5 (PMC13179193; doi:10.1007/s00380-025-02640-5)
Supplement: Supplementary file 1 — Supplementary Table 1. Variables extracted from DPC database. Table listing variables available to be extracted from DPC database for this study. Some coded columns did not have a decoding scheme and could not be accessed or utilised for this study. [file 380_2025_2640_MOESM1_ESM.pdf]

Online Resource

Supplementary Table 1. Variables available from DPC database

Variable	Explanation / Comments
**Inpatient number of admitted hospital**	Number of inpatients of the hospital at which the patient was admitted
**Total medical cost**	Total medical cost of stay in Japanese Yen
**ADL on admission**	Unable to retrieve coding scheme
**ADL on discharge**	Unable to retrieve coding scheme
**Age**	Age of patient at admission
**Ambulance transport**	Unable to retrieve coding scheme
**ICD-10 code of diagnosis on admission**	Diagnosis at admission
**ICD-10 code of comorbidity on admission 1**	Comorbidities diagnosed at admission, up to four diagnoses
**ICD-10 code of comorbidity on admission 2**	Comorbidities diagnosed at admission, up to four diagnoses
**ICD-10 code of comorbidity on admission 3**	Comorbidities diagnosed at admission, up to four diagnoses
**ICD-10 code of comorbidity on admission 4**	Comorbidities diagnosed at admission, up to four diagnoses
**ICD-10 code of Diagnosis after Admission 1**	Any additional diagnoses after admission during hospital stay
**ICD-10 code of Diagnosis after Admission 2**	Any additional diagnoses after admission during hospital stay
**ICD-10 code of Diagnosis after Admission 3**	Any additional diagnoses after admission during hospital stay
**ICD-10 code of Diagnosis after Admission 4**	Any additional diagnoses after admission during hospital stay

**ICD-10 code of Main Diagnosis of Admission**	Main diagnosis looked after during admission
**Diagnosis Procedure Combination code**	Unique insurance code for patient
**ICD-10 code of Diagnosis requiring the most medical resources**	Illness or disease that required the most medical resources in terms of cost
**ICD-10 code of diagnosis requiring the second most medical resources**	Illness or disease that required the second most medical resources in terms of cost
**Death Within 24 hours**	1 = death within 24 hours of admission, 0 = no death within 24 hours of admission
**Height (cm)**	Height in centimeters
**Hospital Dummy Code**	Unique code for hospital
**Department Code**	Department code of attending physician
**Net length of hospital stay (days)**	Total length of hospital stay in days
**Number of admissions**	Unable to retrieve coding scheme
**First Procedure Performed**	First procedure performed after admission
**Second Procedure Performed**	Second procedure performed after admission
**Third Procedure Performed**	Third procedure performed after admission
**Fourth Procedure Performed**	Fourth procedure performed after admission
**Fifth Procedure Performed**	Fifth procedure performed after admission
**Outcome**	Numeric value from 1 to 9. 6 = death due to main diagnosis, 7 = death due to diagnosis other than main diagnosis
**Patient Dummy Code**	Unique code for patient
**SAVR status**	1 = received SAVR, 0 = did not receive SAVR
**Sex**	1 = Male, 2 = Female
**Smoking index**	Unable to retrieve coding scheme

**TAVR status (before 2018)**	1 = received TAVR, 0 = did not receive TAVR
**TAVR status (after 2018)**	2 = received TAVR, 0 = did not receive TAVR
**Urgency**	Unable to retrieve coding scheme
**Weight (kg)**	Weight in kilograms
**Days to first Procedure**	Days from admission to first procedure performed
**Days to second Procedure**	Days from admission to second procedure performed
**Days to third Procedure**	Days from admission to third procedure performed
**Days to fourth Procedure**	Days from admission to fourth procedure performed
**Days to fifth procedure**	Days from admission to fifth procedure performed
**Hospital type**	Type of hospital - primary, secondary, tertiary, specialist center
**Year of hospitalisation**	Year of hospitalisation

Article title: Nationwide Trends and In-Hospital Outcomes of Surgical versus Transcatheter Aortic Valve Replacement in Japan: A Real-World Analysis Using Administrative Data

Journal name: Heart and Vessels

Author names: Yoon Kyoung Kim, Eiki Nagaoka (corresponding author), Kiyotoshi Oishi, Mikayo Toba, Kiyohide Fushimi, and Tomoyuki Fujita

Affiliation of the corresponding author: Department of Cardiovascular Surgery, Institute of Science Tokyo

E-mail address of the corresponding author: nagaoka.cvsg@tmd.ac.jp
